# Regulation of vascular endothelial growth factor expression by homeodomain-interacting protein kinase-2

**DOI:** 10.1186/1756-9966-27-22

**Published:** 2008-07-21

**Authors:** Rosa Puca, Lavinia Nardinocchi, Gabriella D'Orazi

**Affiliations:** 1 Department of Experimental Oncology, Molecular Oncogenesis Laboratory, Regina Elena Cancer Institute, 00158, Rome, Italy; 2 Department of Oncology and Neurosciences, University "G. d'Annunzio", 66013, Chieti, Italy

## Abstract

**Background:**

Homeodomain-interacting protein kinase-2 (HIPK2) plays an essential role in restraining tumor progression as it may regulate, by itself or within multiprotein complexes, many proteins (mainly transcription factors) involved in cell growth and apoptosis. This study takes advantage of the recent finding that HIPK2 may repress the β-catenin transcription activity. Thus, we investigated whether HIPK2 overexpression may down-regulate vascular endothelial growth factor (VEGF) levels (a β-catenin target gene) and the role of β-catenin in this regulation, in order to consider HIPK2 as a tool for novel anti-tumoral therapeutical approaches.

**Methods:**

The regulation of VEGF expression by HIPK2 was evaluated by using luciferase assay with VEGF reporter construct, after overexpression of the β-catenin transcription factor. Relative quantification of VEGF and β-catenin mRNAs were assessed by reverse-transcriptase-PCR (RT-PCR) analyses, following HIPK2 overexpression, while β-catenin protein levels were evaluated by western immunoblotting.

**Results:**

HIPK2 overexpression in tumor cells downregulated VEGF mRNA levels and VEGF promoter activity. The VEGF downregulation was partly depending on HIPK2-mediated β-catenin regulation. Thus, HIPK2 could induce β-catenin protein degradation that was prevented by cell treatment with proteasome inhibitor MG132. The β-catenin degradation was dependent on HIPK2 catalytic activity and independent of p53 and glycogen synthase kinase 3β (GSK-3β) activities.

**Conclusion:**

These results suggest that VEGF might be a target of HIPK2, at least in part, through regulation of β-catenin activity. These findings support the function of HIPK2 as tumor suppressor and hypothesise a role for HIPK2 as antiangiogenic tool in tumor therapy approaches.

## Background

Homeodomain interacting protein-kinase 2 (HIPK2) has been initially identified as corepressor for various homeodomain-containing transcriptional regulators [1]. In the last ten years, HIPK2 has been found to regulate transcription, apoptosis, cell growth, and development, acting both as transcriptional co-repressor and as kinase, through its interaction with a variety of functional proteins [reviewed in ref. [2]]. HIPK2 phosphorylates substrates such as oncosuppressor p53 for activation of its apoptotic function [3,4] or promotes proteasomal degradation of proteins such as MDM2 or CtBP, repressing their antiapoptotic activity [5,6]. It has been shown that Axin forms a ternary complex with HIPK2 and p53, activating p53-dependent transcription and apoptosis [7]. In Wnt signalling, Axin interacts with many components of the pathway, including the adenomatous polyposis coli (APC) tumor suppressor, the serine/threonine kinases casein kinase Iα (CKIα) and glycogen synthase kinase 3β (GSK3β), and β-catenin [8-10]. This complex promotes the degradation of β-catenin through multiple, hierarchical phosphorylation events that, once β-catenin is phosphorylated at Ser-37 and Ser-33 by GSK3β, is recognized by β-transducing repeat-containing protein (β-Trcp) and targeted for proteasomal degradation [11].

One essential element of the Wingless-Wnt signalling pathway is β-catenin, a potent oncogene whose accumulation has been implicated in tumorigenesis in a wide variety of human cancers [12]. β-catenin can be regulated by many biochemical mechanisms, not yet completely understood [13]. In most cases, Wnt/β-catenin pathway is activated by a mutation in APC tumor-suppressor; in many remaining cases, mutations in β-catenin itself at sites of GSK3β phosphorylation lead to β-catenin cytoplasmic accumulation and activation as transcription factor to induce the expression of several target genes, including *c-myc*, *cyclin D1*, *uPAR*, *c-jun*, and *fra-1 *[14-17], involved in cell growth.

Among the β-catenin target genes is vascular endothelial growth factor (VEGF) [18], a potent inducer of angiogenesis both *in vivo *and *in vitro *[19]. Tumor progression is often dictated by increased vascularity following VEGF up-regulation. Thus, due to its role in tumor angiogenesis VEGF is overexpressed in a wide variety of human cancers [20]. The inhibition of VEGF expression has been shown to decrease tumor size in nude mice and inhibit tumor angiogenesis [21]. These findings underline the effort is undertaken to study the regulation of the signalling pathways involved in tumor angiogenesis in an attempt to propose effective multiple-target strategies for the prevention and treatment of human cancers.

These findings, along with a recent study showing that HIPK2 represses the transcription of the β-catenin target cyclin D1 [22], prompted us to investigate the influence of HIPK2 on VEGF expression in tumor cells and the involvement of β-catenin in this regulation.

## Methods

### Cell cultures and reagents

Human lung adenocarcinoma H1299 and human breast cancer MCF7 cell lines were cultured in RPMI-1640 (GIBCO-BRL, Life Technology, Grand Island, NY, USA), human embryonic kidney 293 cells were grown in Dulbecco's modified Eagle's medium (DMEM, GIBCO-BRL), all supplemented with 10% heat-inactivated fetal bovine serum (GIBCO-BRL) plus glutamine and antibiotics in humidified atmosphere with 5% CO_2 _at 37°C. Proteasome inhibitors MG132 (Biomol, Research Laboratories, Plymouth Meeting, PA, USA) was prepared as 50 mM stock in DMSO, stored at -20°C and diluted into the medium at 2.5 μM for 6 h. The treatment with 30 mM LiCl (Sigma Chemical Company, Saint Louis, MO, USA) was for 16 h.

### Transfection, plasmids, and transactivation assay

Transient transfection was carried out using the N,N-bis-(2-hydroxyethyl)-2-amino-ethanesulphonic acid-buffered saline (BBS) version of the calcium phosphate procedure [23]. The amount of plasmid DNA was equalized in each sample by supplementing with empty vector. The expression vectors used in this study were: Flag-HIPK2, Flag-K221R [3]; human HA-β-catenin and mutant HA-S33Y-β-catenin [24] (a kind gift of Avri Ben-Ze'ev, The Weizmann Institute of Science, Rehovot, Israel); the synthetic TOPFlash luciferase reporter (Upstate, Lake Placid, NY, USA), highly specific for Wnt/β-catenin signaling, that contains only LEF1/TCF binding sites; and the human VEGF-luc promoter reporter (kindly provided by C. Gaetano, IDI, IRCCS, Rome, Italy). Transfection efficiency was normalized with the use of a co-transfected β-galactosidase plasmid. Luciferase activity was assayed on whole cell extracts and the luciferase values were normalized to β-galactosidase activity and protein content. At least three independent experiments were performed in duplicate.

### RNA extraction and RT-PCR analysis

Cells were harvested in TRIzol Reagent (Invitrogen) and total RNA was isolated following the manufacturer's instructions. The first strand cDNA was synthesized according to the manufacturer's instructions (Moloney murine leukemia virus reverse transcriptase kit, Applied). Semi-quantitative RT-PCR was carried out by using HOT-MASTER Taq (Eppendorf) with 2 μl cDNA reaction and genes specific oligonucleotides under conditions of linear amplification. The sequence of the primers used for RT-PCR was as follow: human β-catenin forward: 5'-GAAAATCCAGCGTGGACAATGGCTACT-3' and reverse 5'-ACC-ATAACTGCAGCCTTATTAACC-3'; human VEGF forward: 5'-CCTGGTGGACATCTT-CCAGGAGTA-3'; and reverse: 5'-TCACCGCCTCGGCTTGTC-ACA-3'. The VEGF amplification leads to doublets representing the 165 and 121 VEGF isoforms, as previously shown [25]. DNA products were run on 2% agarose gel and visualized by ethidium bromide using UV light. Densitometric analysis was applied to quantify mRNA levels. Data presented are representative of at least three independent experiments.

### Immunoblotting

Total cell extracts were prepared by incubating at 4°C for 30 min in lysis buffer (50 mM Tris-HCl, pH 7.5, 50 mM NaCl, 5 mM EDTA, 150 mM KCl, 1 mM dithiothreitol, 1% Nonidet P-40) plus a mix of protease inhibitors (Sigma Chemical Company). Proteins were then separated by SDS-PAGE and blotted onto nitrocellulose (Bio-Rad). The membranes were probed with a primary antibody followed by a secondary antibody conjugated with horseradish peroxidase. The antibodies used were: rat monoclonal anti-HA (Roche Diagnostics); rabbit polyclonal anti-cyclin D1 (Santa Cruz Biotechnology, kindly provided by M. Crescenzi, ISS, Rome, Italy); mouse monoclonal anti-tubulin (Sigma Chemical Company); rabbit polyclonal anti-β-catenin (Santa Cruz Biotechnology), rabbit polyclonal anti-phospho-GSK3α/β (Cell Signaling Technology, Inc., Danvers, MA, USA), and mouse monoclonal anti-GSK3β (Santa Cruz Biotechnology). Immunoreactivity was detected with the ECL chemoluminescence reaction kit (Amersham Corp., Arlington Heights, IL, USA). Data presented are representative of at least three independent experiments.

### Statistical analysis

Continuous variables were analyzed by the Student *t *test. Data are expressed as mean ± SD. A value of *p *≤ 0.05 was considered statistically significant.

## Results

### HIPK2 decreased VEGF mRNA levels in tumor cells

**Figure 1:**
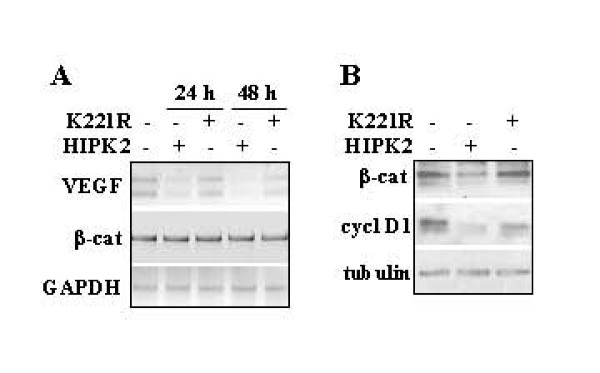
**Effect of HIPK2 on VEGF and β-catenin levels**. (A) MCF7 cells were transfected with HIPK2 or the K221R kinase defective mutant and 24 and 48 h after transfection VEGF and β-catenin (β-cat) mRNA levels were evaluated by reverse transcriptase-PCR (RT-PCR) analysis. GAPDH was used as loading control. One representative experiment from three independent experiments was shown. (B) Cells were transfected as in (A) and 36 h after transfection cell extracts were subjected to SDS-PAGE and immunoblotting was performed with anti-β-catenin and anti-cyclin D1 (cycl D1) antibodies. Anti-tubulin was used as protein loading control. One representative experiment from three independent experiments was shown.

High levels of VEGF expression have been associated with tumor angiogenesis and therefore with tumor progression in a wide variety of human cancers [20]. To determine whether HIPK2 affected VEGF expression, MCF7 cells were transfected with HIPK2 or K221R kinase defective expression vectors. Levels of VEGF mRNA were analysed by RT-PCR 24 h and 48 h post-transfection. As shown in Figure 1A, levels of VEGF mRNA were downregulated by HIPK2 but not by K221R mutant. Interestingly, the β-catenin mRNA levels were not affected by HIPK2 overexpression (Figure 1A) while the β-catenin protein levels decreased after HIPK2 but not after K221R overexpression (Figure 1B). In agreement with β-catenin downregulation, also the β-catenin transcription activity was impaired, as suggested by decreased cyclin D1 levels after HIPK2 overexpression (Figure 1B).

These results indicate that HIPK2 overexpression decreased both VEGF mRNA and β-catenin protein levels and β-catenin transcription activity. The possible link between HIPK2 and β-catenin-mediated transcriptional regulation of VEGF was next evaluated.

### HIPK2 suppressed the β-catenin-mediated transcriptional activation of VEGF

**Figure 2:**
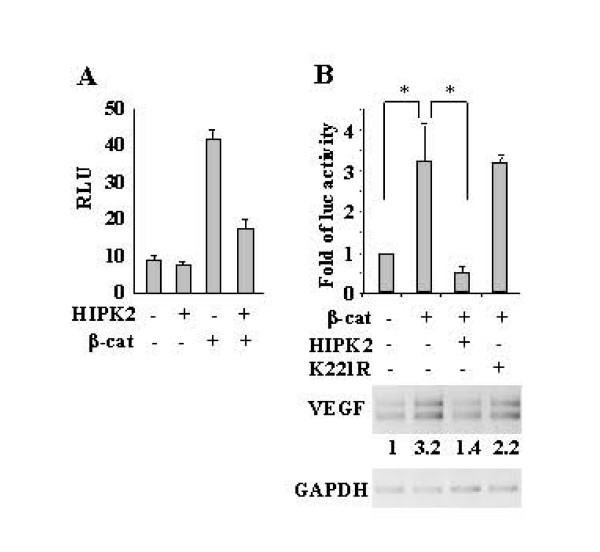
**Effect of HIPK2 on β-catenin-induced VEGF transcription**. (A) Transcriptional activation of reporter plasmid expressing luciferase under the optimal TCF-responsive element (TOPFlash) was evaluated following co-transfection of H1299 cells with β-catenin and HIPK2 expression vectors. Thirty-six h after transfection, cells were assayed for luciferase activity. Results are expressed as Relative Luciferase Units (RLU) and represent the mean ± SD from three independent experiments performed in duplicate. *p < 0.001 *versus *β-catenin alone. (B, upper panel) H1299 cells were co-transfected with VEGF-luc reporter construct along with β-catenin and HIPK2 or K221R mutant, for detection of luciferase activity as above. The data shown as fold of luciferase activity represent the mean ± SD from three independent experiments performed in duplicate. *p < 0.001. (B, lower panel) VEGF mRNA levels were determined by RT-PCR analysis. GAPDH was used as loading control. One representative experiment from three independent experiments was shown. Densitometric analysis of VEGF levels was performed and normalized values to GAPDH mRNA levels were indicated.

The effect of HIPK2 on β-catenin-mediated transcription activation was first evaluated on artificial TCF/LEF-1 luciferase reporter plasmid. To this end, H1299 cells were co-transfected with TOPFlash reporter and a combination of HIPK2 and β-catenin expression vectors. As shown in Figure 2A, HIPK2 strongly suppressed the β-catenin-induced luciferase activity driven from TOPFlash reporter, as recently shown [22]. Next, we evaluated whether HIPK2 could suppress the activity of the VEGF reporter induced by β-catenin. To this aim, H1299 cells were co-transfected with VEGF-luc reporter along with combinations of β-catenin and HIPK2 or K221R expression vectors. As shown in Figure 2B (upper panel), β-catenin induced VEGF luciferase activity that was significantly suppressed by HIPK2 co-expression, while the K221R mutant failed to do so. In agreement with these findings, VEGF mRNA levels induced by β-catenin were strongly inhibited by HIPK2 co-transfection (Figure 2B, lower panel).

Altogether, these data show that HIPK2 inhibited β-catenin-mediated VEGF transcription and that HIPK2 catalytic activity was likely involved in this regulation.

### Effect of HIPK2 on β-catenin proteasomal degradation

**Figure 3:**
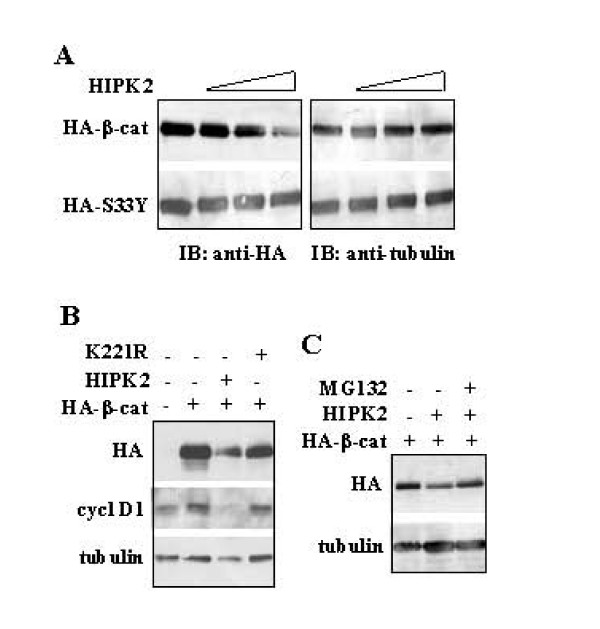
**HIPK2 is involved in β-catenin proteasomal downregulation**. (A) 293 cells were co-transfected with HA-β-catenin or HA-S33Y β-catenin mutant along with increasing amount (1, 3, 5 μg) of HIPK2 expression vector. Thirty-six hours after transfection, total cell extracts were separated on denaturing SDS-PAGE and analyzed by immunoblotting (IB) with anti-HA antibody (to detect β-catenin) and anti-tubulin (as protein loading control). (B, C) 293 cells were transfected with HA-β-catenin and HIPK2 or K221R expression vectors in the presence or absence of the proteasome inhibitor MG132 (2.5 μM) for 6 h and analyzed by immunoblotting with anti-HA (to detect β-catenin), anti-cyclin D1, and anti-tubulin (as protein loading control) antibodies.

Western blot analysis was performed in order to test whether the inhibition of HIPK2-dependent β-catenin activity could depend by β-catenin protein destabilization, as HIPK2 can regulate protein turnover through phosphorylation-targeted/proteasomal degradation [5,6]. To this aim, 293 cells were co-transfected with constant level of HA-tagged β-catenin or the S33Y mutant β-catenin, with increasing amounts of HIPK2 protein. As shown in Figure 3A, HIPK2 promoted reduction of β-catenin levels in a concentration-dependent manner. In contrast, HIPK2 did not reduce the levels of the S33Y (Figure 2A), a mutant β-catenin, which is refractory to phosphorylation by GSK3β and proteasomal degradation [24]. To evaluate whether HIPK2 catalytic activity was involved in β-catenin down-regulation, 293 cells were co-transfected with β-catenin and HIPK2 or K221R expression vectors. As shown in Figure 3B, HIPK2, but not K221R, efficiently reduced β-catenin levels and likely its transcription activity, as also suggested by cyclin D1 downregulation (Figure 3B). Finally, HIPK2-induced degradation of co-transfected β-catenin was reverted by cell treatment with the proteasome inhibitor MG132 (Figure 3C).

**Figure 4:**
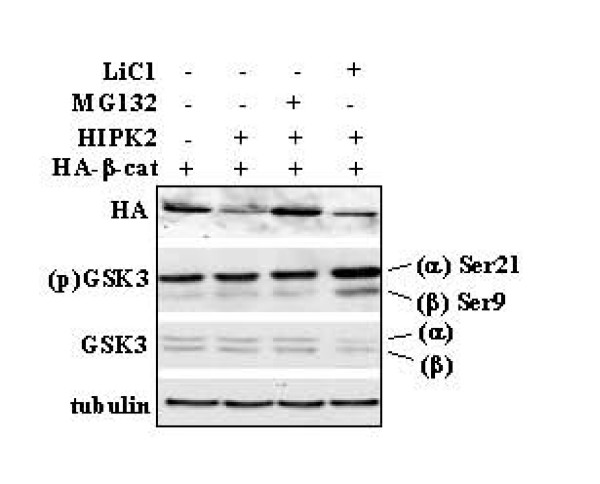
**HIPK2-mediated β-catenin proteasomal downregulation independent of p53 and GSK3β activities**. H1299 (p53/null) lung adenocarcinoma cells were transfected with HA-β-catenin and Flag-HIPK2 expression vectors, in the presence or absence of the proteasome inhibitor MG132 (2.5 μM) for 6 h, or the specific GSK3β inhibitor LiCl (30 mM for 16 h). Thirty-six h after transfection, total cell extracts were analysed by Western immunoblotting with anti-HA (to detect β-catenin), anti-phospho-GSK3 (to detect Ser21 and Ser9 phosphorylations of respectively GSK3α and GSK3β), and anti-GSK3 (to detect total GSK3α and GSK3β proteins) antibodies. Anti-tubulin was used as protein loading control.

Since HIPK2 is a potent activator of p53 function [3,4] and it has been reported that activated p53 can promote degradation of β-catenin [24], we next examined the β-catenin protein levels by HIPK2 in p53/null cells; moreover, we also examined whether the β-catenin downregulation required the activity of GSK3β. To this aim, we co-expressed β-catenin and HIPK2 in H1299 cells (p53/null) in the presence or absence of the proteasome inhibitor MG132 or the specific GSK3β inhibitor LiCl [26]. As shown in Figure 4, HIPK2 strongly reduced β-catenin levels that were rescued by MG132 cell treatment, as shown before; interestingly, LiCl treatment did not block the ability of HIPK2 to lower β-catenin levels despite GSK3β was strongly phosphorylated in serine 9 and therefore inactive [26,27].

These data suggest that HIPK2 down-regulated β-catenin levels through proteasomal degradation system, an effect dependent on HIPK2 catalytic activity and independent of p53 and GSK3β activities.

## Discussion

The results presented in this study revealed a novel target of HIPK2 oncosuppressor function that is VEGF, and strengthened the role of HIPK2 as transcriptional repressor of β-catenin-function. We showed that HIPK2 down-regulated both endogenous VEGF mRNA levels and the VEGF levels induced by β-catenin overexpression, in different tumor cells, highlighting interplay between HIPK2-mediated β-catenin regulation and VEGF expression. Thus, we have found that HIPK2 might induce β-catenin proteasomal degradation and inhibit its transcription activity.

The tumor progression is often dictated by increased vascularity following VEGF up-regulation [19]. Thus, due to its role in tumor angiogenesis VEGF is overexpressed in a wide variety of human cancers [19,20]. However, angiogenesis is not restricted to the advanced stages of cancer but can also be observed early in premalignant stages of tumor development. Thus, many molecular mechanisms deregulated in cancer cells might act as VEGF inducers, creating a challenge in tumor therapy for blocking VEGF production and starve tumors [i.e., see ref. [28]]. Among the mechanisms that can up-regulate VEGF in cancer is the Wnt pathway and the β-catenin transcription factor [18]. Therefore, inhibiting the output of the Wnt pathway remains a goal for therapeutical intervention for many tumors [12].

Increasing evidence indicates that HIPK2 is able to modulate the transcription activity of a growing number of transcription factors involved in cell growth and apoptosis [reviewed in ref. [2]]. Recently, β-catenin activity has been identified as target of HIPK2 transcriptional repression [22], thus it was shown that HIPK2 suppresses β-catenin-mediated activation of cyclin D1, controlling cell proliferation. In agreement with this report, here we found that HIPK2 might downregulate the β-catenin target VEGF. The β-catenin transcription activity was inhibited by HIPK2-mediated proteasomal degradation, moreover we showed that HIPK2 but not its catalytic inactive mutant was able to downregulate β-catenin protein levels, suggesting that HIPK2 catalytic activity might be involved in β-catenin phosphorylation/degradation. HIPK2 has been shown also by our studies to phosphorylate substrates for either activating their apoptotic function, such as oncosuppressor p53 [3,4], or promoting proteasomal degradation of antiapoptotic proteins such as MDM2 or CtBP [5,6]. Thus, our effort is since several years to study the molecular mechanisms underlying the role of HIPK2 in restraining tumor progression in p53-dependent and independent ways. However, whether HIPK2 acts directly or indirectly inside multiprotein regulatory complexes, on β-catenin phosphorylation and therefore activity remains to be elucidated.

Collectively, the data presented in this study indicated that HIPK2 could repress the β-catenin transcription activity and downregulate VEGF expression in tumor cells. Constitutive activity of β-catenin can exert both proliferative and antiapoptotic effects [29] as well as VEGF expression favours tumor progression [18-21]. Hence, the downregulation of β-catenin by HIPK2, also in p53/null cells, is likely to contribute to the antiproliferative effects of HIPK2. However, the existence of multiple different pathways for both β-catenin and VEGF regulation may provide a fail-safe mechanism in case one of the components along either of the pathways becomes inactivated. Many different mechanisms can contribute to VEGF up-regulation and in this regard we have recently found that HIPK2 can regulate the HIF-1α-induced VEGF expression, inhibiting tumor angiogenesis (L.N. and G.D.O. unpublished results). In conclusion our findings support the potential role of HIPK2 as oncosuppressor and might give important contributions for the development of novel combinatory anti-tumoral therapeutical approaches focused to deal with tumor angiogenesis.

## Competing interests

The authors declare that they have no competing interests.

## Authors' contributions

RP carried out cell transfection, immunoblotting analysis, and luciferase assays, LN contributed to cell transfection, cell treatments, RT-PCR, and densitometric analyses. GDO supervised experimental work and wrote the manuscript. All authors read and approved the final manuscript.
